# Tactile localization accuracy at the low back

**DOI:** 10.3758/s13414-024-02843-4

**Published:** 2024-02-08

**Authors:** Simon Pratt, Benedict M. Wand, Dana A. Hince, Mervyn J. Travers, Lee Schneider, Sara Kelly, William Gibson

**Affiliations:** 1 School of Health Sciences, The University of Notre Dame Australia, Fremantle, WA Australia; 2 Institute for Health Research, The University of Notre Dame Australia, Fremantle, WA Australia

**Keywords:** Tactile localization, Superficial schema, Tactile acuity, Body representation

## Abstract

Localizing tactile stimulation is an important capability for everyday function and may be impaired in people with persistent pain. This study sought to provide a detailed description of lumbar spine tactile localization accuracy in healthy individuals. Sixty-nine healthy participants estimated where they were touched at nine different points, labelled in a 3 × 3 grid over the lumbar spine. Mislocalization between the perceived and actual stimulus was calculated in horizontal (x) and vertical (y) directions, and a derived hypotenuse (c) mislocalization was calculated to represent the direct distance between perceived and actual points. In the horizontal direction, midline sites had the smallest mislocalization. Participants exhibited greater mislocalization for left- and right-sided sites, perceiving sites more laterally than they actually were. For all vertical values, stimulated sites were perceived lower than reality. A greater inaccuracy was observed in the vertical direction. This study measured tactile localization for the low back utilizing a novel testing method. The large inaccuracies point to a possible distortion in the underlying perceptual maps informing the superficial schema; however, further testing comparing this novel method with an established tactile localization task, such as the point-to-point method, is suggested to confirm these findings.

## Introduction

The ability to localize the site of tactile stimulation is a basic requirement for successfully interacting with and understanding the world around us and our place in it. To understand the mechanisms behind tactile localization, it is helpful to consider the postural schema (Head & Holmes, 1911; Longo, 2015; Longo et al., 2010), the superficial schema (Longo, 2015; Longo et al., 2010), the body model, and postural priors (Tamè et al., 2019). The postural schema represents the appreciation of body posture and is associated with *where* the body is in space (Berlucchi & Aglioti, 2010; Head & Holmes, 1911; Longo et al., 2010). The superficial schema operates as a linking function, connecting localization on somatotopic maps and localization of touch on the body (tactile localization) (Longo, 2015; Longo et al., 2010; Longo et al., 2015). This schema reflecting the localization of a stimulated spot on the skin was first proposed by Head and Holmes (1911). The effective interaction of our body with its surroundings is in part due to tactile localization, assisted from our postural schema informing the “where” of our body parts in space, and the superficial schema informing the “where” of external stimuli touching our skin, with the combined process referred to as *tactile spatial remapping* (Longo et al., 2015). The concept of tactile spatial remapping therefore proposes that, in order to localize a tactile stimulus, information on body posture such as proprioception must be combined with tactile information informing the location on the skin of a stimulus (Longo et al., 2015). The body model assists with this process by providing information on body size and shape (Longo & Haggard, 2010; Tamè et al., 2019). Postural priors, which develop from frequent touch while adopting particular stored body configurations, are hypothesized to interact with the postural schema to produce fast localization of touch in space (Tamè et al., 2019). Spatial priors, stored representation concerning the most plausible location of touch in visual space, have also been linked to assisting with tactile spatial remapping (Tamè et al., 2019). Recently, it has been suggested that the complex processing of touch may also be related to the source of touch and to high-level categorical information about body parts (Tamè & Longo, 2023). Furthermore, functional representations of touch such as somatosensory processing of different body parts or the functional role of the stimulus or action have also been proposed to influence the processing of touch (Tamè & Longo, 2023).

Pain has been shown to affect tactile function, for example, people with chronic low back pain exhibit a greater lumbar two-point discrimination distance threshold (11.74 mm or 9.49 mm if excluding high risk of bias studies) (Adamczyk et al., 2018a), reduced lumbar graphesthesia accuracy (Wand et al., 2010), and higher levels of mislocalization to light touch or pinprick over the back (Wand et al., 2013) when compared to healthy controls. Furthermore, models of persistent pain suggest a causative role for deficits in sensory precision (Moseley & Vlaeyen, 2015; Wand et al., 2022) and a recent high-quality clinical trial demonstrated clinically relevant and sustained improvements in outcome for people with persistent low back pain from a treatment program that included tactile localization training over the lumbar spine (Bagg et al., 2022).

A significant number of studies have evaluated lumbar tactile precision in healthy individuals by assessing two-point discrimination thresholds (Adamczyk et al., 2016; Beaudette et al., 2016; Catley et al., 2014; Catley et al., 2013; Falling & Mani, 2016; Flaherty & Connolly, 2014; Luomajoki & Moseley, 2011; Nishigami et al., 2015; Stanton et al., 2013; Wand et al., 2010). Tactile localization ability has received much less attention in healthy individuals (Adamczyk et al., 2016; Cholewiak et al., 2004; Wand et al., 2013), and extensive testing of tactile localization and in-depth quantification of localization accuracy over the whole low back has yet to be studied.

The aim of this study was to investigate how accurate healthy individuals are in estimating where they were touched at nine points over the low back, comparing perceived and actual measures in horizontal (x) and vertical (y) directions, and for a derived hypotenuse (c) direct distance between the perceived and actual measures. As participants were required to also be aware of where their back was in space to locate the stimulus, tactile spatial remapping could be argued to be involved with the tactile localization task. Obtaining preliminary data is key to interpreting this measure in patients with chronic low back pain and evaluating outcomes in people undergoing localization training.

## Methods

### Experimental design

We conducted a cross-sectional observational study within a university research laboratory. The study had approval from the Institutional Human Research Ethics Committee (Reference Number: 017188F). All participants provided signed informed consent and all procedures conformed to the tenets of the Declaration of Helsinki.

### Participants

A consecutive sample of 69 healthy volunteers was recruited by advertisement and word of mouth from The University of Notre Dame Australia and the local community between February 2018 and March 2019. Inclusion criteria included: aged 18–60 years; currently low back pain free; no history of low back pain lasting more than 24 h within the last 6 months; no low back pain requiring medical attention within the last 2 years; no other significant musculoskeletal pain (> 1/10); able to stand in a stable position for up to 1 h; proficient in written and spoken English; and able to provide informed consent. Exclusion criteria included: known body perception difficulties (e.g., body dysmorphic disorder; anorexia; vestibular disorder); non-correctable visual impairment; unstable balance in standing; any current neurological, musculoskeletal, or widespread pain disorder; any significant existing medical condition and any large tattoo over the back, which could not be suitably erased with photoshop (part of exclusion criteria for a concurrent study not reported here).

### Apparatus

**Fig. 1:**
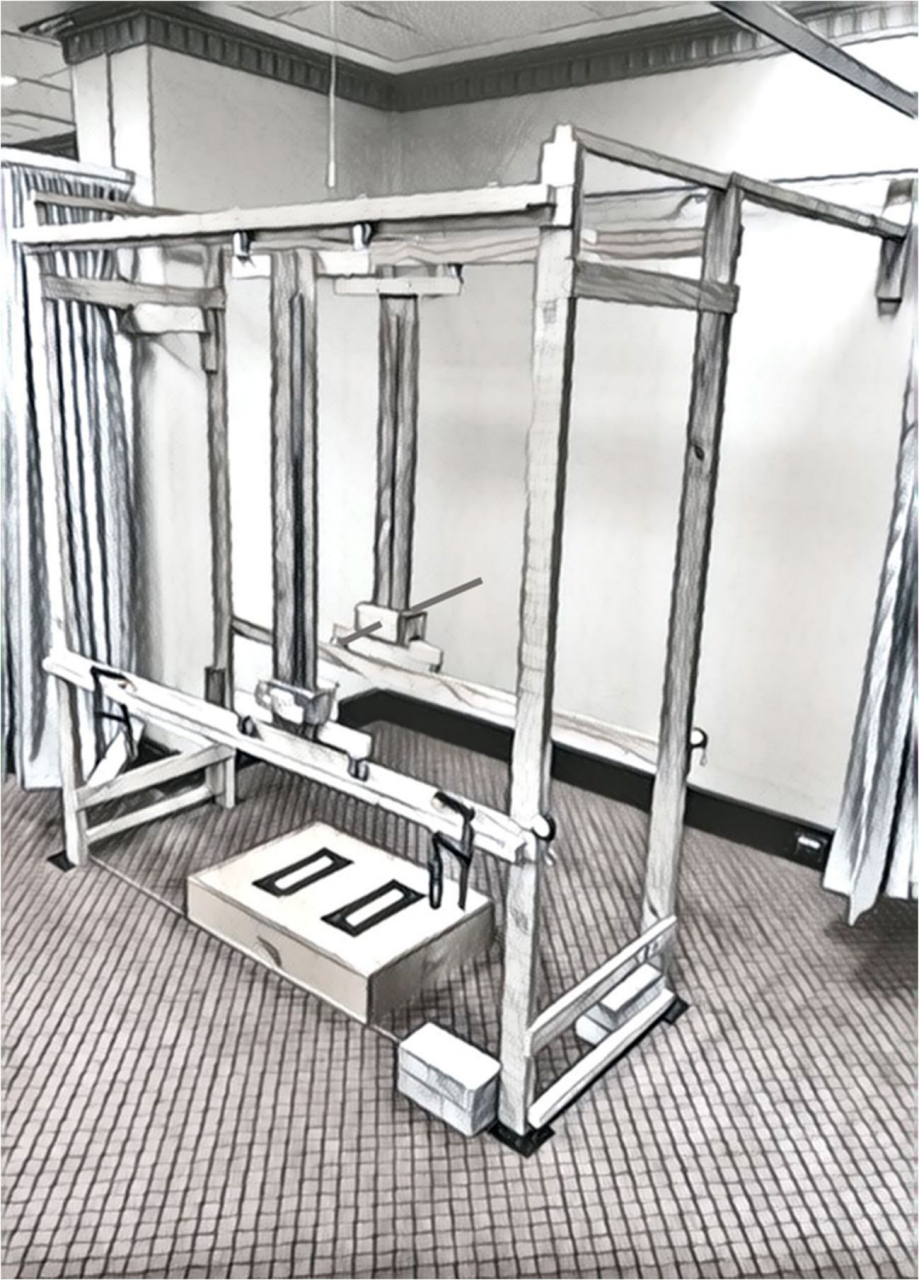
The back representation frame (BRF)

The testing device, the back representation frame (BRF) (Fig. 1), is a three-dimensional wooden frame 183.3 cm in length, 63.4 cm wide, and 202.0 cm in height anchored to the wall posteriorly and laterally. The reliability of this device has been previously reported (Pratt et al., under review). On the posterior part of the frame a cylindrical metal pointer (5 mm diameter) mounted on wheels can be moved on a track in the vertical (x) and horizontal (y) planes (Fig. 1). Two measurement rulers are fixed to the frame, enabling the x and y co-ordinates of the pointer position to be recorded by an assessor. Participants are unable to see the pointer or the measurement ruler when standing in the frame. On the anterior aspect of the frame, in front of the participant, a matching pointer is also mounted on tracks, which can be moved by participants in both horizontal and vertical directions. Similarly fixed rulers allowed the x and y co-ordinates of the anterior pointer to be measured by a second assessor.

This device allows actual and perceived values to be measured with one assessor recording the coordinates of the actual tactile stimulus on the low back posteriorly, and a second assessor recording the coordinates of the participant’s estimate of the site of this stimulus by noting the position of the anterior pointer.

### Experimental procedures

A demonstration on the use of the BRF was provided to all participants, the testing procedure was explained, and any questions were answered. Participants were offered a practice trial of the task before formal testing; however, not every participant performed a practice trial, as some participants did not elect to practice after seeing this task demonstrated.

### Standardized testing position

At the commencement of each testing session, the diagonal dimensions of the frame were checked to ensure consistency across all testing sessions. A 13.5-cm high box was positioned within the frame and its position checked against markings of a string line attached to the frame. Participants stood on the box with their feet hip-width apart, and in a neutral position with toes facing forward.

The participant pointer on the anterior part of the frame was initially positioned 10 cm (or as close as possible to this) from the most anterior point of the participant’s stomach. To set the starting position for testing, participants were asked to hold the pointer at a self-selected comfortable height, and the participant’s shoulder was then placed in 30° abduction. Participants returned to this starting position after each repetition. Participants were advised to ensure their arm did not touch their body when moving the pointer and to keep their free arm in slight abduction away from the side of their back with testing. This was to reduce any external tactile feedback. If participants accidentally touched their body when moving the pointer, the measure was disregarded, and the trial repeated.

**Fig. 2:**
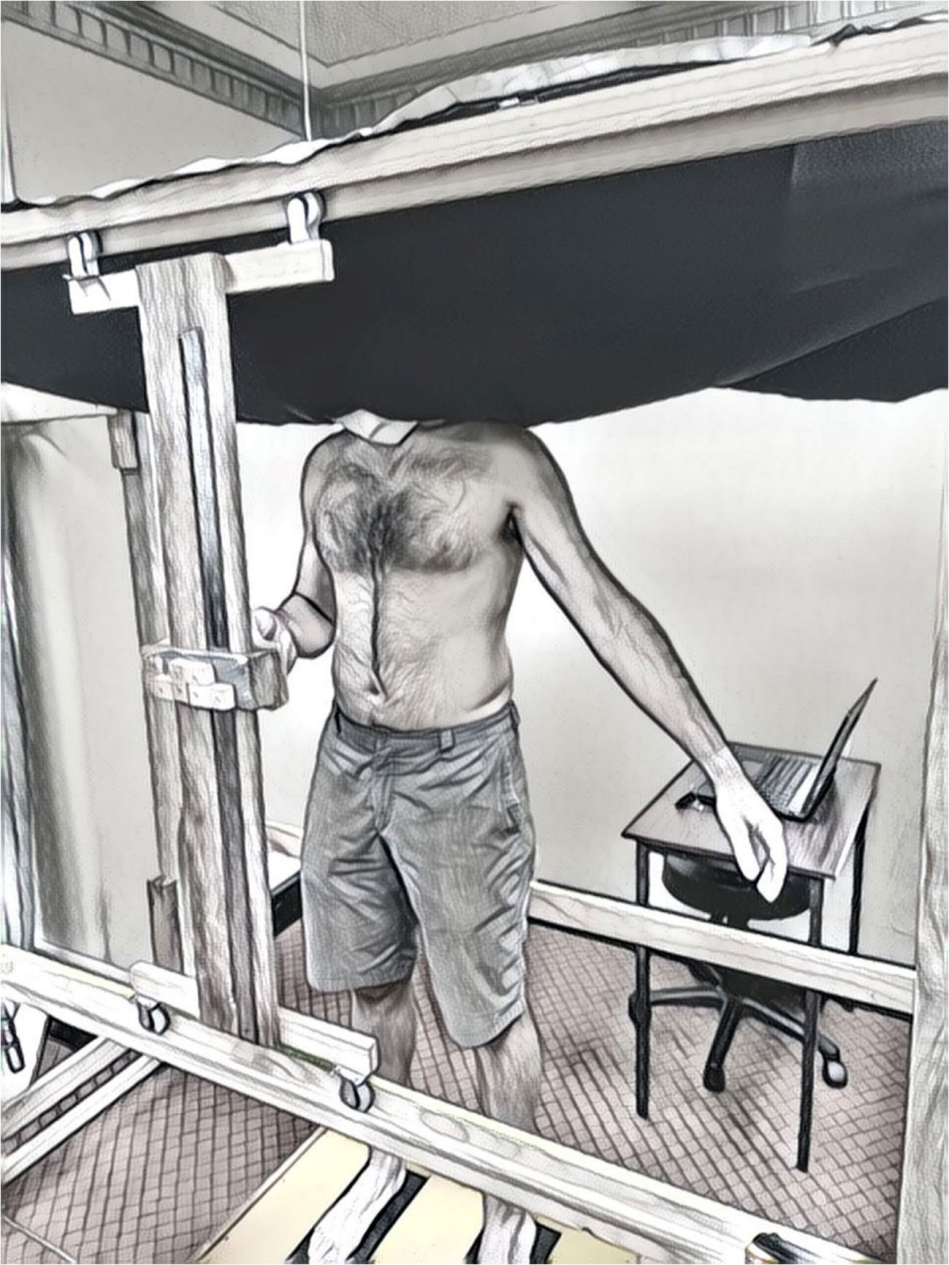
Standardized testing position

For testing, participants wore peripheral vision-blocking goggles and a black sheet was comfortably pegged into place around the participant’s neck to prevent them from seeing their body or the anterior measurement rulers (Fig. 2). When moving the pointer and estimating the perceived tactile stimuli location, participants closed their eyes; however, to reduce postural sway when testing, participants were instructed to open their eyes once satisfied with their perceived position.

### Tactile localization task

**Fig. 3:**
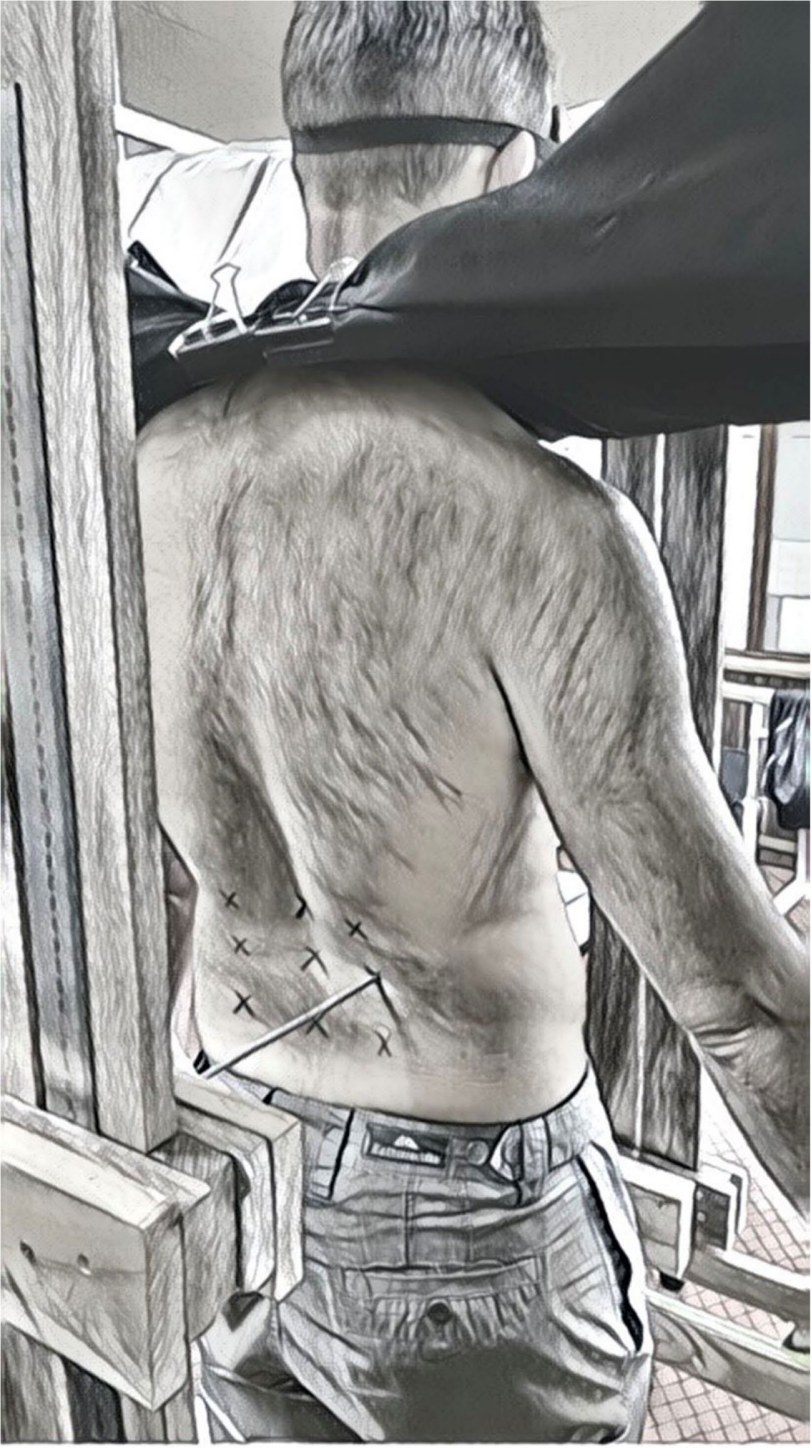
Tactile localization stimulation sites with stimulus being applied

Initially, participants lay face down on a plinth, and the spinous processes of L1, L3 and L5 were marked with a felt-tipped pen. The widths of the participants’ low back at these three levels were measured using a set of metal calipers (Mentone Educational Anthropometer Measuring Set), with precision of 1 mm. Six additional stimulation sites were marked on either side of the L1, L3 and L5 points by calculating 40% of the caliper measured width at each level, and marking each point 40% to the left and right of the midline, thus forming a 3 × 3 grid over their lumbar spine, with the top row, left to right, as stimulation sites 1, 2, 3; the middle row, left to right, as stimulation sites 4, 5, 6; and the bottom row, left to right, as stimulation sites 7, 8, 9 (Fig. 3).

With the patient standing within the frame in the standardized position, tactile localization was then assessed. The tactile stimulus was applied by the first assessor (standing behind the patient) via a blunt-tipped rigid metal rod pushed through the centre of the posterior pointer until it touched the skin at the pre-selected landmark (Fig. 3). The stimulus was applied to the first blanching of the skin for 3 s, with the x and y values for each stimulus recorded as the actual location. No verbal or visual information was available to the participant regarding which landmark was stimulated. Following removal of the stimulus, participants were instructed to move the centre of their pointer to the anterior correlate of where they perceived the touch on their low back to have been. For this anterior correlate, participants were advised to envision an imaginary line going through their body from their low back to the front at the point where the external tactile stimulus was applied. Once satisfied with their localization attempt, the x and y values of the participants’ pointer position were recorded by a second assessor in front of the participant as the perceived location. This assessor was unaware of the values recorded by the first assessor.

### Order of testing

Each of the nine stimulation sites were assessed four times. The midline stimulation sites were assessed twice with each hand, the left hand was used for all stimulation sites on the left side and the right hand was used for all stimulation sites on the right side. This was done to prevent participants gaining feedback by the arm touching the body as they crossed midline. Random allocation was first used to determine if tasks involving the left hand or tasks involving the right hand would be performed first. A computer-generated random sequence was then used to determine the order of testing with that hand (four repetitions for the three lateral stimulation sites and two repetitions for the three midline stimulation sites). Testing was then completed using the alternate hand and order determined by a separate computer-generated random sequence.

### Mislocalization variable derivation

The horizontal (x) and vertical (y) mislocalization values at each stimulation site were calculated by subtracting the actual x and y values of the applied stimulus (recorded by the first assessor) from each pair of the corresponding x and y values of the participant’s perceived position of the stimulus (recorded by the second assessor). A finding of ‘0’ for the x or y values indicated perfect accuracy in that plane. A positive value for horizontal mislocalization indicated the perceived stimulation site was to the left of the actual stimulus, and a negative value indicated the perceived stimulation site was to the right of the actual stimulus. A positive value for vertical mislocalization indicated the perceived stimulation site was higher than the actual stimulus, and a negative value indicated the perceived stimulation site was lower than the actual stimulus. A further derived ‘c’ or hypotenuse mislocalization was calculated from the horizontal and vertical mislocalizations, and represented the straight-line distance between the perceived and actual site of stimulation. This distance was treated as a scalar value, ignoring the direction associated with this derived value.

## Data analysis

Statistical analysis was performed using the Statistical Package for Social Sciences (SPSS) version 24 (IBM Corporation, New York, USA), and STATA- V17 (StataCorp. 2017, Stata Statistical Software: Release 17, College Station, TX: StataCorp LLC). The demographic profile of participants was summarized with means and standard deviations (SDs) for continuous data and frequency/percentages for binary data. A p-value < 0.05 was considered evidence for a difference.

The horizontal, vertical and hypotenuse mislocalizations were analyzed using separate linear mixed models for each outcome and included a crossed random effect for place and level, nested within each participant. Preliminary models tested the association of place (left, midline, right), level (top, middle, bottom rows) and the interaction between the two with each outcome, but only the factors that returned a Wald χ2 p-value < 0.05 are included in the models reported below. Estimated means are reported with 95% confidence intervals. All final models met the assumption of homoscedasticity, normality of residuals and normality of random effects. Individual data points were reviewed if the standardized residual from the model was > 5 standard deviations from the mean. The value for that trial was deemed missing if it was impossible or had evidence of human error in the context of values recorded for the same trial type.

No a priori sample size calculation was carried out for these analyses as the data in this current study was collected as part of a larger study. Despite this analysis not being outlined here, compared with a low back pain group of the same size at the alpha = 0.05 level, 60 participants per group provided over 80% power to detect a between-group difference in horizontal direction bias at the midline of 17.5 mm (SD = 34 mm).

## Results

Ninety-eight participants were screened for eligibility; however, for the following reasons, 29 were excluded: history of low back pain within the last 6 months (12); low back pain requiring medical attention, or lumbar spine surgery, within the last 2 years (1); known body perception difficulties (1); other musculoskeletal or widespread pain presentations (12); significant existing medical condition (2); tattoo over the back (1). Sixty-nine healthy participants were therefore enrolled in the study, comprising 34 males (49.3%) and 35 females (50.7%) with a combined average age of 29.3 years (SD = 9.5) and an average body mass index of 23.9 (SD = 3.8). Sixty-three (91.3%) participants were right-handed, and six (8.7%) were left-handed. All 69 participants completed all tasks; however, 0.3% (7/2,477) of actual x trials, and 0.2% (4/2,480) of actual y trails were deemed data entry errors based on residual analyses. There were no missing data for perceived x or y trials.

**Fig. 4:**
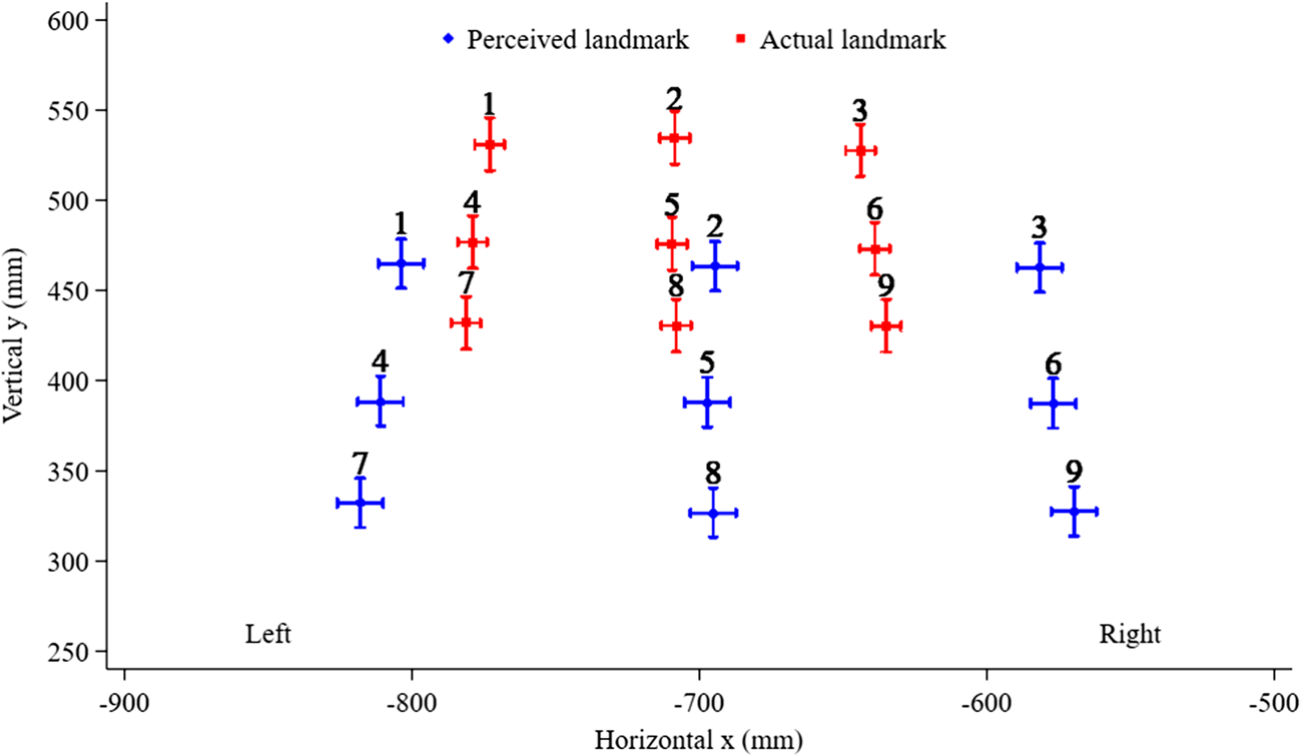
Perceived versus actual x–y locations and corresponding error bars for stimulation sites 1–9 of the tactile localization task. The top row, left to right, corresponds to stimulation sites 1, 2, 3; the middle row, left to right, corresponds to stimulation sites 4, 5, 6; and the bottom row, left to right, corresponds to stimulation sites 7, 8, 9

**Fig. 5:**
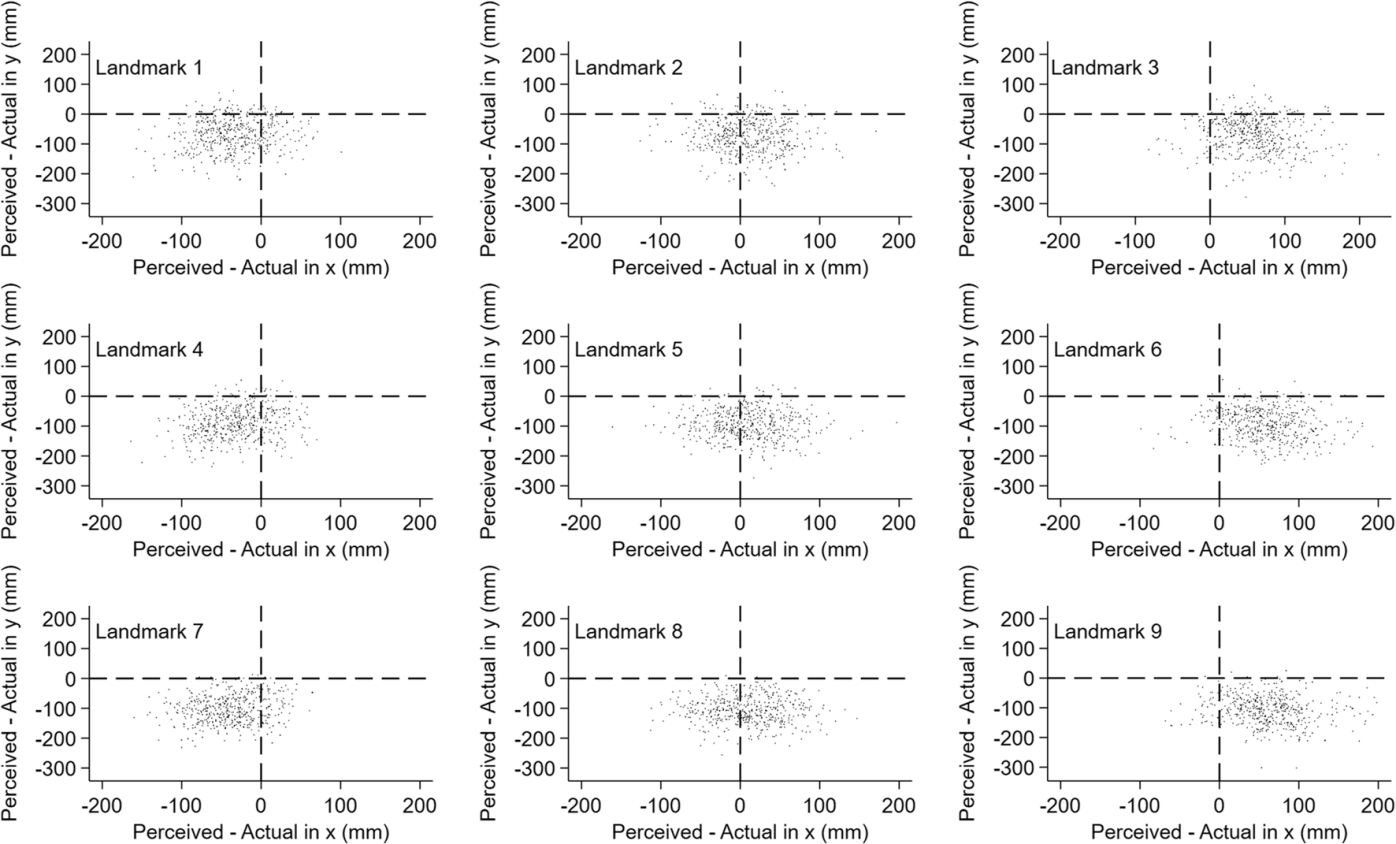
Individual estimations from each participant at each point for the tactile localization task

Figure 4 illustrates the perceived and actual x and y locations for stimulation sites 1–9, highlighting the downward shift of perceived locations relative to the actual site of stimulation, the lateral shift of lateral stimulation sites, and the rightward shift of the midline stimulation sites relative to the actual site of stimulation. The x and y co-ordinate mean values and corresponding error bars are presented in Fig. 4, and the associated standard deviations are in Table 1, highlighting the consistent underestimation of the perceived vertical (y) values and the higher vertical mislocalization values compared to corresponding horizontal mislocalization values. Individual estimations from each participant at each point are presented in Fig. 5.

**Table 1:** Observed perceived, actual and mislocalization (perceived minus actual) means (mm) and associated standard deviation (SD) (mm) values for stimulation sites 1–9 in the horizontal (x) and vertical (y) directions

	Left lateral stimulation sites				Midline stimulation sites				Right lateral stimulation sites			
		Perceived	Actual	Mislocalization		Perceived	Actual	Mislocalization		Perceived	Actual	Mislocalization
x	1	803.7 (31.8)	773.0 (24.7)	30.7 (33.9)	2	694.5 (26.0)	708.6 (20.3)	-13.9 (27.0)	3	581.6 (36.2)	644.0 (20.1)	-62.4 (39.1)
	4	810.9 (34.1)	778.9 (24.3)	32.0 (34.6)	5	697.3 (25.8)	709.5 (20.2)	-12.0 (24.8)	6	576.9 (36.5)	638.9 (19.9)	-62.1 (38.0)
	7	818.0 (36.2)	781.1 (24.9)	36.9 (34.1)	8	695.2 (23.7)	708.1 (19.8)	-12.8 (24.9)	9	569.6 (37.9)	634.9 (21.1)	-65.4 (41.9)
y	1	464.8 (62.2)	531.0 (62.3)	-66.2 (44.8)	2	463.4 (59.9)	534.6 (62.8)	-71.2 (48.0)	3	462.7 (62.1)	527.5 (62.6)	-65.0 (53.7)
	4	388.4 (54.5)	476.8 (62.3)	-88.6 (42.0)	5	388.0 (58.1)	475.8 (62.2)	-87.8 (42.7)	6	387.5 (55.9)	473.0 (61.4)	-85.6 (47.2)
	7	332.3 (58.5)	431.9 (60.6)	-99.6 (41.4)	8	326.7 (54.5)	430.4 (59.8)	-103.8 (41.2)	9	327.5 (56.9)	430.3 (61.1)	-102.8 (46.6)

### Horizontal (x) direction

Only the place (left, middle, or right) of the stimulation sites assessed was related to the degree of mislocalization in the horizontal direction (effect of place, χ2 [2] = 462.1, p < 0.001). As shown in Fig. 4, mislocalization for left lateral stimulation sites was further lateral to the left (33.2 mm, 95% CI = 25.6 to 40.8, p < 0.001). Midline stimulation sites exhibited a rightward bias (-12.9 mm, 95% CI = -20.5 to -5.4, p = 0.001) and the right lateral stimulation sites a substantially larger rightward bias (-63.3 mm, 95% CI = -70.9 to -55.7, p < 0.001). The right lateral stimulation site’s mislocalization was on average 50.3 mm greater than that for the midline (95% CI = 41.5 to 59.2, p < 0.001).

### Vertical (y) direction

In the vertical direction, only the level (top, middle, or bottom row) of the stimulation sites assessed was related to the degree of mislocalization (effect of level, χ2 [2] = 140.8, p < 0.001). As shown in Fig. 4, mislocalization in the vertical direction increased from the top (-67.5 mm, 95% CI = -77.5 to -57.5, p < 0.001), to the middle (-87.3 mm, 95% CI = -97.3 to -77.3, p < 0.001), to the bottom level (-102.1 mm, 95% CI = -112.1 to -92.1, p < 0.001). The mislocalization at the middle was 19.8 mm (95% CI = 14.1 to 25.5, p < 0.001) greater than at the top level, and 14.8 mm (95% CI = 9.0 to 20.5, p < 0.001) greater at the bottom compared with the middle level.

### Hypotenuse (c) value

The degree of mislocalization for the hypotenuse value was dependent upon both the level (effect of level, χ2 [2] = 117.0, p < 0.001) and the place of the stimulation sites assessed (effect of place, χ2 [2] = 28.2, p < 0.001). As shown in Table 2, the hypotenuse value mislocalization increased from midline to right (15.7 mm, 95% CI = 9.0 to 22.3, p < 0.001), and from left to right (15.7 mm, 95% CI = 9.0 to 22.4, p < 0.001), which reflected the large rightward bias for the right lateral stimulation sites. Hypotenuse value mislocalization also increased when moving lower down the low back, with 14.0 mm greater mislocalization at the middle compared with the top level (95% CI = 9.1 to 18.9, p < 0.001), and 13.0 mm greater at the bottom compared to the middle level (95% CI = 8.1 to 17.9, p < 0.001), which reflected the increasing vertical direction mislocalization when moving from top to middle to bottom levels.

**Table 2:** Hypotenuse (c) value predicted mean mislocalization (perceived minus actual) (mm)

	Left			Midline			Right		
Level	Predicted Mean Mislocalization (mm)	95% CI	*p*-value	Predicted Mean Mislocalization (mm)	95% CI	*p*-value	Predicted Mean Mislocalization (mm)	95% CI	*p*-value
Top	87.1	77.6 to 96.6	< 0.001	87.1	77.6 to 96.7	< 0.001	102.8	93.3 to 112.3	< 0.001
Middle	101.1	91.6 to 110.6	< 0.001	101.1	91.6 to 110.7	< 0.001	116.8	107.3 to 126.3	< 0.001
Bottom	114.1	104.6 to 123.7	< 0.001	114.2	104.6 to 123.7	< 0.001	129.8	120.3 to 139.4	< 0.001

## Discussion

The aim of this study was to investigate the accuracy of tactile localization over the low back in healthy individuals. We used a specially designed back representation frame to compare perceived stimulation site coordinates to actual stimulation site coordinates in horizontal, vertical and hypotenuse directions for nine points at the low back. For horizontal values, we found that midline stimulation sites had the smallest mislocalization error, consistent with previous findings at the lumbar spine using the point-to-point test at the L3 level (Adamczyk et al., 2016). For the midline sites, participants demonstrated only a small rightward bias. For stimulation sites on the left and right**,** participants perceived the stimulations to be substantially more lateral than they actually were; the rightward bias was still reflected in this with a greater lateral error on the right compared to the left.

For all vertical values, participants perceived the stimulation to be lower than it actually was. This finding may be analogous to the distal bias seen in previous studies investigating localization accuracy in the limbs (Longo et al., 2015; Mancini et al., 2011; Margolis & Longo, 2015). On average, as the level of the stimulation site became lower on the lumbar spine, the value of the vertical mislocalization became larger, a phenomenon also seen to a small extent moving distally from the forearm to the wrist (Longo, 2017), but in contrast to the increased error seen moving more proximally at the hand (Margolis & Longo, 2015). Interestingly, vertical mislocalizations were larger than corresponding horizontal mislocalizations, indicating a higher degree of inaccuracy in the vertical direction; indeed, the relative accuracy of localization of midline sites in the horizontal direction is not at all replicated in the vertical direction. These larger errors in the vertical direction are in line with previous findings of higher spatial acuity of touch *across* relative to *along* the limbs (Margolis & Longo, 2015; Weber, 1834/1996). In the low back, the higher degree of accuracy in the horizontal direction could be hypothesized to be due to clearer reference points to anchor off – a fairly accurate midline as a potential categorical boundary (Nicula & Longo, 2021) and very clear lateral borders – compared to non-equivalence of boundaries in the vertical direction, with much more “room” to make a mistake vertically.

The derived hypotenuse mislocalization increased midline to right, and left to right, reflective of the large rightward and lateral bias for the right stimulation sites, and increased moving from top to middle to bottom levels, reflective of the increasing vertical direction mislocalization. These findings suggest that the lumbar spine might be perceived as being closer to the ground than reality, and more stretched in the horizontal and vertical directions compared to reality, at least with respect to localizing tactile input. This stretched perception has been demonstrated in tactile localization investigations at the forearm (Trojan et al., 2006), though compressed perceptions have also been noted (Trojan et al., 2006, 2009). Tactile localization tasks at the hand (Longo et al., 2015) and hand and forearm (Longo, 2017) have also demonstrated overestimation in the medio-lateral direction when mapping perceived shape and size. Interestingly, these findings are in some agreement with our investigation of perceived size and shape of the back in the absence of sensory input, which suggests that the implicit body model of the back demonstrates a rightward shift as well as an overestimation of waist and hip width, though, in contrast, we found evidence that the held representation of the lowest part of the back may be shrunken in the vertical direction (Pratt et al., under review).

A range of methodologies have been employed to assess tactile localization and a number of body areas assessed. Most investigations have focused on the limbs, and these studies have shown that localization errors are a common finding in healthy individuals (Cicmil et al., 2016; Culver, 1970; Longo, 2017; Mancini et al., 2011; Manser-Smith et al., 2018; Manser-Smith et al., 2019; Margolis & Longo, 2015; Mattioni & Longo, 2014; Omar & Samuel, 2020; Steenbergen et al., 2012, 2013; Trojan et al., 2006, 2009; Yoshioka et al., 2013). Localization accuracy is thought to be a manifestation of distortions in underlying perceptual maps (Mancini et al., 2011), where perceptual maps refer to the somatotopic maps of stimulation patterns as subjectively perceived on the body surface (Trojan et al., 2006). Localization errors for the upper limb in school-aged children has been reported as 9–17 mm (Omar & Samuel, 2020), whereas previous studies involving participants over the age of 19 years have reported localization error values of around 27 mm (Trojan et al., 2009) for the forearm. Culver (1970) reported localization errors of 37.9 to 114.5 mm at the hand; however, these appear to be the sum of errors in x and y directions, and so comparison is difficult. These values, however, are in stark contrast to the mislocalizations seen in our study, with hypotenuse values ranging from 87 mm to 130 mm. Looking toward the body, whether or not the body is actually seen has also been shown to modulate tactile localization (Medina et al., 2018). The greater inaccuracies seen at the back may represent a reflection of estimating localization for a body part that is not routinely visualized. Greater inaccuracies could also be linked to relative body size, with the low back a relatively larger body part, compared to the hand, for example. Previous studies have reported localization error as a percentage of actual body size, discovering underestimation of finger length of approximately 39% (Mattioni & Longo, 2014), overestimation in the horizontal direction of the hand of between 55% (Mattioni & Longo, 2014) and 75% (Longo et al., 2015), and an overestimation error of distances of approximately 12% in the vertical direction and 115% in the horizontal direction when considering the hand, wrist and forearm regions overall (Longo, 2017). As part of a separate study, we calculated actual width at the narrowest part of the waist for the same participants, and based on this actual width, horizontal mislocalizations varied between 4.6% and 22.3% of body size.

From a functional perspective, tactile localization requires us to know where on the body part we have been touched and where the body part is in space, a process referred to as *tactile spatial remapping* (Longo et al., 2015), and this is how we have assessed localization in this study. The evidence in studies outlined above appears to suggest tactile localization of stimuli on the skin utilizes a distorted representation. However, it may be this does not adequately represent the underlying mechanisms through which this phenomenon is created. Longo et al. (2015) demonstrates position sense of the body part in question can contribute heavily to these distortions. Observed distortions seen using a methodology whereby the participant must use knowledge of the (occluded) body part in space (as per our current study) may in fact be more reflective of position sense as opposed to tactile localization distortions (Longo et al., 2015). This introduces a degree of complexity to interpretation of our results. Our study could be argued to engage tactile spatial remapping; that is, we required participants to localize stimuli on the skin *and* localize the body in external space. Thus, our findings may be due to distortions in localizing the body in external space (position sense), as opposed to localization of touch on the skin (tactile localization). Therefore, in our study, while we did not explicitly produce perceptual maps derived from our mislocalizations, any inferences drawn from the current results should be considered in light of these complexities.

There are relatively few studies investigating tactile localization over the low back. Cholewiak et al. (2004) assessed vibrotactile localization at 12 sites around the circumference of the trunk, including the spine, and at two different levels: the waist at a level 25 mm above the navel, and approximately 100 mm higher, over the lower margin of the rib cage. Localization at the midline points of the spine and navel were virtually perfect, and sites adjacent to these midline points were better localized than those at the sides, which correspond to the lateral stimulation sites in the current study, and had an approximate 65–70% accuracy rate (Cholewiak et al., 2004). This higher accuracy at the midline, compared to lateral sites, is consistent with results from the current study. The fact the spine is on the midline, and has bilateral cortical representation, may play a role in this higher accuracy (Cholewiak et al., 2004). Spatial localization has been suggested to improve near reference points such as joints (Plaisier et al., 2020), including the spine (Cholewiak et al., 2004; Plaisier et al., 2020). These reasons may explain the higher accuracy observed in our study with horizontal midline sites. However, caution needs to be applied with adopting this explanation as a later study found localization accuracy to vibrotactile stimuli was reduced near the spine compared to more peripherally at the thoracic spine (Jouybari et al., 2021), and more specifically, at the lower thoracic spine (Hoffmann et al., 2018); furthermore, our study did not find greater accuracy vertically at the midline. Wand et al. (2013) asked healthy participants to lay prone on an examination table and presented them with a picture of the posterior view of the body with the trunk and thighs divided into 12 zones. Individual zones were stimulated with light touch or pinprick multiple times in random order and the participants were asked to nominate which zone had been stimulated. This study suggested good tactile localization capacity with 75% of participants making no errors; however, this methodology did not enable localization error to be quantified precisely and required participants to reference the touch in relation to a picture of the body, rather than their own body. The point-to-point task was developed to more precisely quantify tactile localization over the lumbar spine (Adamczyk et al., 2016). This task involves touching participants at the lumbar spine at the L3 spinous process and a further two points, horizontally separated by 5 cm and 10 cm from this midline point, and asking participants to show with a pen on their lumbar region where on the skin they perceived it to have been stimulated (Adamczyk et al., 2016). Data from healthy individuals shows similar values that were on average much lower than the current study. Mean mislocalization scores for the two examiners were lowest at midline (19.5 mm), and larger for the lateral sites (27.4 mm at 5cm from the midline; 26.8 mm at 10cm from the midline) (Adamczyk et al., 2016). Whilst this study only used three points at one level of the spine, one on midline and two points randomly assigned to one side of the body, our study employed points at three separate levels to both sides of the spine, further encompassing the low back region, and investigating both vertical and horizontal direction. This expansion of further points across both sides of the spine and multiple levels may partly explain the larger inaccuracies recorded in the current study, whilst recognizing a difference in study methodologies also exists.

Though different to the methodology used here, other investigations of tactile function have noted differences that mirror findings of greater error in the vertical direction. A study presenting vibrotactile stimulation to the thoracolumbar spine found distances felt longer in the vertical direction compared with the horizontal direction (Plaisier et al., 2020). Differences in perceived distance have been argued to occur due to different densities and shapes of receptor fields (Longo & Haggard, 2011) and cortical representations over different skin regions, with a further rescaling process of distorted cortical representations to preserve size constancy (Taylor-Clarke et al., 2004). This may help to explain the differences observed in horizontal and vertical mislocalizations in the current study. At the lower thoracic spine, participants demonstrated higher accuracy from vibrotactile stimuli for horizontal compared with vertical presentation (Hoffmann et al., 2018), with a further study reporting distances from pressure stimuli presented vertically were overestimated relative to distances oriented horizontally (Nicula & Longo, 2021). Additional research at the thoracic spine reported a significantly greater number of vertical localization errors with vibrotactile and force stimulation, and reduced accuracy for trials presented along the vertical axis in a direction discrimination task when using vibrotactile stimulation (Jouybari et al., 2021). These findings at the thoracic spine share similarities with our study where mislocalizations were greater in the vertical direction. However, in contrast, at the upper back (centre of scapula), distances from pressure stimuli oriented across the body width were overestimated compared to along body height (Nicula & Longo, 2021).

Tactile function has been widely investigated in people with persistent pain (Kuttikat et al., 2018; Menten et al., 2022; Stanton et al., 2013; Trojan et al., 2019), including people with low back pain (Adamczyk et al., 2018a, b, 2019; Luomajoki & Moseley, 2011; Moseley, 2008; Spahr et al., 2017; Wand et al., 2010; Wand et al., 2013; Wang et al., 2020, 2022). Largely, these studies suggest that persistent pain is characterised by a loss of sensory precision, most commonly through the observation of larger two-point discrimination thresholds in people in pain (Adamczyk et al., 2018a; Luomajoki & Moseley, 2011; Moseley, 2008; Stanton et al., 2013; Wand et al., 2010). Tactile discrimination training, which primarily includes training of tactile localization, has been applied to a number of pain conditions, including low back pain (Barker et al., 2008; Flor et al., 2001; Morone et al., 2012; Moseley et al., 2008; Ryan et al., 2014), and may offer some benefit to this population (Kälin et al., 2016). More complex interventions for low back pain have been developed that include tactile localization training as part of a comprehensive care package (Wand et al., 2022), and early promising results reported for this type of approach (Wälti et al., 2015; Wand et al., 2011) have been replicated in a large placebo-controlled trial (Bagg et al., 2022). Currently, there is a mismatch between the assessment of tactile dysfunction (two-point discrimination) and the type of training employed (tactile localization). The development of a tool that enables a comprehensive evaluation of tactile localization over the low back will offer further insight into the nature of tactile dysfunction in low back pain, and may provide data that enriches current sensory training approaches. Sequential assessment of both two-point discrimination and tactile localization in people under care for low back pain may also offer insights into the mechanism of action of treatment approaches that include sensory training. The collection of the preliminary data presented here is the first step in this important research program.

There are some limitations to the present study that need to be recognized. Firstly, considering the process of tactile process remapping, our findings may be due to distortions in localizing the body in external space, instead of due to localization of touch on the skin. Secondly, participants were required to engage in coordinate transformation and take the location of touch on their back and then transform the location to the space in front of them; thus, it is unclear what influence such a transformation process may have on results as opposed to distortions in body representation. Future studies could engage participants in performing two tasks, the tactile localization method in this current study, and a localization judgement task on a back template with the same grid presented, similar to a study involving the hand by Mancini et al. (2011). Performing these two tasks would investigate if biases observed were related to touch or a more general spatial bias, and would enable analysis into individual differences in bias across tasks. Thirdly, to check if the lateral bias seen in the perceived landmark location may be influenced by the hand used, we did check the midline results by hand used, and there is a clear rightward bias when the right hand is used; there is also some evidence of a leftward bias with the left hand, though this is very small, making interpretation difficult. Fourthly, the tactile localization measure was taken as the last task in a 2- to 2.5–h testing session, and participant fatigue may have been a factor. Future studies could look to utilize this method in a stand-alone assessment session. Fifthly, for ease of comparison with other literature, this study could have recorded body size of width and height at the location of the nine stimulation sites to enable calculation of localization error as a percentage of body size. Variables such as how the size of the area being stimulated compares to error values, and the number of repeated measures, could be examined.

## Conclusion

This study employed a novel methodology to investigate the accuracy of tactile localizations in the lumbar spine, with participants referencing localizations to their actual body. Considering perceived and actual horizontal values, participants demonstrated the smallest mislocalization error, and only a small rightward bias with midline stimulation sites, left and right stimulation sites were perceived more laterally than they actually were, particularly on the right side, again reflecting a rightward bias. For all vertical values, participants perceived the stimulation site lower than it actually was, which may be considered a distal bias. This error also increased as the stimulation moved inferiorly. A higher degree of inaccuracy in the vertical direction was observed, with vertical mislocalization values larger than corresponding horizontal mislocalization values at all stimulation sites. Overall, tactile localization of the low back demonstrated overestimation in the horizontal and vertical directions, pointing to a possible distortion in underlying perceptual maps informing the superficial schema, although further work is required to clarify this. Changes in tactile acuity have been demonstrated in people with low back pain; utilizing the methodology described here in people with low back pain may give a richer understanding of tactile dysfunction in this population as well as providing insight into the process of clinical improvement in those undertaking sensory precision training.

## Data Availability

Code used in statistical analysis may be shared with other researchers if a formal request for information is made.

## Declarations

### Data availability/Open practices statement

Data may be shared with other researchers if a formal request for information is made. None of the experiments were preregistered.
